# Influence of antagonist tooth on mandibular implant positioning during surgery among Indians

**DOI:** 10.6026/97320630019474

**Published:** 2023-04-30

**Authors:** Surendran Sundaram, Subhashree Rohinikumar, Abhinav Rajendra Prabhu, Thiyaneswaran Nesappan, Vishnu Priya Veeraraghavan, Rajalakshmanan Eswaramoorthy

**Affiliations:** 1 Department of Implantology, Saveetha Dental College and Hospitals, Saveetha Institute of Medical and Technical Sciences, Saveetha University, Chennai 600077, India; 2 Department of Biochemistry Saveetha Dental College and Hospitals, Saveetha Institute of Medical and Technical Sciences, Saveetha University, Chennai 600077, India; 3 Department of Biomaterials, Centre of Molecular Medicine and Diagnostics (COMManD), Saveetha Dental College and Hospitals, Saveetha Institute of Medical and Medical and Technical Sciences, Saveetha University, Chennai 600077, India

**Keywords:** Dental implant, deviation, mandible, cone beam computed tomograph

## Abstract

A retrospective radiographic analysis of cone beam computed tomographic radiographs of 42 patients who had undergone dental implant therapy at the department of implantology, Saveetha Dental College Hospitals, India. The mean angular deviation was 3.17 ° in the anterior, 1.6° in the premolar and 0.81° in the molar region. Data shows that free hand placement could be done with minimal deviation taking the opposing dentition as a guide.

## Background:

Dental implants have become the go to procedure to replace missing teeth, with progressively increasing availability of data on long term implant survival and success [1]. Suboptimal placement done by diverting from the prosthetically driven order may result in many untoward outcomes starting from biological complications to complete prosthetic failure, various factors have been attributed, most commonly being the position of adjacent teeth in the edentulous site, restricted mouth opening, resorbed alveolar and the surgeon's dexterity [2]. The favourable implant position is not a single point but a zone; this aids the development of proper emergence profiles and to maintain the bone levels around the endosseous fixture. In aesthetically important zones the proper 3D implant positioning is of prime importance [5], Long-term benefits of an appropriate restorative-driven implant placement [3][4], include favourable aesthetics and function, as well as ideal occlusion and masticatory forces distribution[6]. There have been an increased use of guides to achieve this, but these devices increase the cost and make the procedure more tedious and cannot be used in all situations [7][8] [9]. Therefore, it is of interest to document the influence of antagonist tooth on mandibular implant positioning during surgery among Indians.

##  Materials and Methods:

## Selection of Subjects:

Approval of the study protocol and ethical clearance were obtained from the Institutional Review Board, Saveetha Dental College Hospitals, Chennai, India and were within the statutory limitations of the Revised Helsinki Declaration of World Health Organization 2013. This study was designed as described elsewhere [10, 11, 12, 13, 14, 15, 16, 17, 18].

## Sample Size:

The sample was calculated considering the mean expected difference and pooled standard deviation obtained from previous literature. The minimum sample size for this pilot study having two groups required to observe the difference (with type I error 5% and power at 80%) was 42 [20].

## Inclusion and Exclusion criteria:

The inclusion criteria included individuals undergoing dental implant surgery where a single root form endosseous implant was placed in mandible with opposing maxillary antagonist - natural unrestored, without super-eruption and without any deviation.

## Measurement of angular discrepancies:

The CBCT pre and post treatment was taken from the radiology repository of the Department of Implantology, Saveetha Dental College. The radiographs were acquired using Carestream CS 9600 the FoV was 8/8, with potential difference of 120 kV at 7 mA, exposure was done for 15 seconds, the obtained data was analysed using CS 3D Imaging v3.10.21 software to measure the angle from the fossa of the maxillary tooth along the long axis to the mandibular ridge, followed by the measuring the fossa of the maxillary tooth to long axis of the implant. The obtained data was then tabulated, and the values were subjected to statistical analysis.

## Statistical Analysis:

Normality of the data was tested using Shapiro - Wilks test, because the data showed normal distribution, parametric tests - paired sample t test was done IBM SPSS Statistical Software version 23.

## Results:

A total of 84 patients in this study, the was no gender disparity and the average age was about 32.3 ± 4.6 years, paired sample t test was done and it was observed that the mean angle noted in the anterior zone (pre-treatment 93.09o ; post treatment 89.92°) premolar zone (pre-treatment 104.73°; post treatment 103.12°) and molar zone (pre-treatment 178.38°; post treatment 177.57°). Angular deviation was calculated to be 3.17° ± 0.92° in the anterior region, 1.61° ± 1.9° in the premolar region and 0.8° ± 0.73° in the molar region (p = 0.001).(Table 1)

## Discussion:

In the age of guided surgery and navigation-based approaches, free hand surgery is still being practised due to the economic reasons and other limiting factors, A well-designed prosthetic plan and implant placement driven by prosthetics can assist the dentist during both the surgical procedure and the final rehabilitation stages [20], thus it becomes imperative that the surgeon should be aware of the existing dentition, and the occlusal pattern prior surgery to place the implant in an optimal position. The importance of correct implant placement also avoids problems related to emergence profiles, aesthetic concerns and hygiene maintenance issues [21]. The acceptability of freehand placed dental implants in clinical practice was assessed based on factors such as the presence of adjacent teeth, the implant quadrant, the number of missing teeth, and the location of the implant site. The study found that the number of missing teeth did not affect implant positioning, while the presence of adjacent teeth, implant quadrant, and location of the implant site had an impact on the direction and angulation deviations of freehand placed implants [22]. The results of a multi-centered clinical trial showed that while the accuracy of guided surgeries in the anterior region was high, it decreased in the distal area due to potential movement of the surgical guide or the need to use an open sleeve design [23]. Numerous studies have attempted to investigate the results of dental implant placement using freehand protocols, and after a 3-year observation period, no statistically significant differences were found between template-guided and freehand placement methods [24].In a similar fashion, freehand and template-guided implant placements were compared in a study that followed up on patients for one year, but no disparities were observed between the two methods [25]. These studies analysed multiple variables, such as implant survival rate, complications, and bone loss around the implant, to compare freehand and template-guided implant placements. A meta-analysis also corroborates the finding that there are no significant differences in implant survival rate, mechanical and biological complications, and marginal bone loss between the two methods [26]. Experience is a significant factor that contributes to implant angulation, especially in free-hand surgery. Single implants are easier to handle, but when it comes to multiple implants, special care is required. During free-hand surgery, practitioners need to consider the risk of approaching the root of an adjacent tooth. A study examining the relationship between the experience of the guide and implant angulation found that practitioners with less than 5 years of experience accounted for 9.3% of cases with 0-degree angulation, while those with 5-10 years and more than 10 years of experience accounted for 37.1% and 53.6%, respectively, of cases with 0-degree angulation [27]. From the above data analysis, it is apparently revealed that the accuracy of freehand placement is optimal especially in the posterior zones, considering that the opposing tooth angulation and occlusal patterns are taken into considerations thus the implant can be positioned in a 3D optimal position.

## Conclusion:

Data shows that using the antagonist tooth (maxillary teeth angulation) for mandibular implant positioning during placement improves placement accuracy with a very mild degree of deviation within acceptable range, given the clinicians experience and other factors that can influence the procedure. On evaluation it is observed that there is a certain minimal degree of deviation between pre-treatment plan and post implant placement and the deviation is minimal within an optimal range.

## Figures and Tables

**Table 1:** Average angular deviation obtained on comparing pre-surgical and post-surgical values

**Tooth**	**Angular Measurements**		**Deviation(degree)**	p*
	Pre-operativen = 42(degree)	Post-operativen = 42(degree)		
Anteriors	93.09± 5.97	89.9± 6.11	3.17± 0.92	.000*
Premolars	104.7± 5.65	103.1± 5.46	1.61± 1.9	.000*
Molars	178.3± 4.53	177.5± 4.48	0.8± 0.73	.000*
